# Pacemaker Created in Human Ventricle by Depressing Inward-Rectifier K^+^ Current: A Simulation Study

**DOI:** 10.1155/2016/3830682

**Published:** 2016-02-21

**Authors:** Yue Zhang, Kuanquan Wang, Qince Li, Henggui Zhang

**Affiliations:** ^1^Biocomputing Research Center, School of Computer Science and Technology, Harbin Institute of Technology, Harbin 150001, China; ^2^School of Physics & Astronomy, University of Manchester, Manchester M13 9PL, UK

## Abstract

Cardiac conduction disorders are common diseases which cause slow heart rate and syncope. The best way to treat these diseases by now is to implant electronic pacemakers, which, yet, have many disadvantages, such as the limited battery life and infection. Biopacemaker has been expected to replace the electronic devices. Automatic ventricular myocytes (VMs) could show pacemaker activity, which was induced by depressing inward-rectifier K^+^ current (*I*_K1_). In this study, a 2D model of human biopacemaker was created from the ventricular endocardial myocytes. We examined the stability of the created biopacemaker and investigated its driving capability by finding the suitable size and spatial distribution of the pacemaker for robust pacing and driving the surrounding quiescent cardiomyocytes. Our results suggest that the rhythm of the pacemaker is similar to that of the single cell at final stable state. The driving force of the biopacemaker is closely related to the pattern of spatial distribution of the pacemaker.

## 1. Introduction

In the heart, the sinoatrial node (SAN) cells are the source of the normal excitation, initiating the heartbeat and control the rhythm [1]. However, the genuine pacemaker, consisting of no more than 10,000 cells, drives the whole heart which contains about 10 billion cells [2]. The failure of the SAN cells causes heart rhythm disorders, leading to syncope, easy fatigability, or circulatory collapse.

The best way to treat these diseases is to implant electronic pacemakers, which have been used clinically for more than half a century with continuous refinement and reduced mortality associated with complete heart block and SAN dysfunction [3]. Nevertheless, there are still many disadvantages: (a) the limited battery life requires replacement at periodic intervals; (b) infection may require removal of the pacemaker; (c) the setting rate is not able to respond to emotion; (d) the device must be tailored to the growth of pediatric patients [4]. Therefore, biopacemaker has been expected to replace the electronic devices due to its ability of recapitulating the main aspects of endogenous SAN.

Three general approaches have been focused to create biopacemakers: (a) introduce special ion channels into cardiomyocytes by gene transfer; (b) express ion channels in noncardiomyocytes and then send them to native myocytes in situ by cell fusion; (c) use embryonic stem cells grown along a cardiac lineage and manifesting the electrophysiological properties of SAN cells [5].

Miake et al. dominant-negatively inhibited the Kir2-encoded ion channel, the inward-rectifier potassium channels, by which nonpacemaking ventricular cells were turned into automatic pacemaking cells [6]. Cell fusion strategies were used by Plotnikov et al. [7] and Cho et al. [8] to create biopacemakers, unfortunately, which did not resemble the natural pacemaking cells. Xue et al. differentiated the embryonic stem cells into cardiomyocytes with pacemaker activity and successfully introduced them directly into the heart of pigs or guinea pigs [9]. Nonetheless, the teratogenic potential and heterogeneity [10] influenced the clinical translation.

A long time will be taken to create a permanent biopacemaker, and the preliminary target is to make a temporary biopacemaker for pacemaker-dependent patients. Once infection occurs, it always needs a complete removal of all the electronic hardware [11]. When the device is removed, the biopacemaker could provide hardware-free chronotropic support during the antibiotic treatment to clear the infection, which typically required about 2 weeks [12]. Accordingly, most subsequent experiments took at least 14-day observation and record. In fact, short-term biopacemakers have been successfully created. Qu et al. produced biopacemakers in the canine hearts by open-chest [13] and transarterial left-sided [14] approaches, however, in which the delivery methods were extremely invasive.

Recently, biopacemaker technology has developed fast. Cingolani et al. [15] first applied the venous catheters to create biopacemaker. The atrioventricular node was ablated to make a complete heart block, and then an adenoviral vector cocktail (KAAA + H2), expressing dominant-negative *I*_K1_ (Kir2.1AAA) and hyperpolarization-activated cation channel (HCN2) genes, was injected into the atrioventricular junctional region through the femoral vein. The suppressed *I*_K1_ unleashed the automaticity of ventricular myocytes (VMs) while the exogenous funny current (*I*_*f*_) simultaneously increased the automatic depolarization in phase 4. Under the dual-gene approach, the significant pacemaker activity was observed over a 14-day period. They concluded that the delivery of KAAA + H2 into atrioventricular junction region induced biopacemaker activity, moreover, which was conducted through the His-Purkinje system.

The lineage reprogramming has been used for converting one cell type into another one. Transcription factors, such as Shox2, Tbx3, Tbx5, and Tbx18, are known as the obvious candidates of embryonic SAN development [16]. Additionally, Tbx18 could downregulate Cx43 but not Cx45 or Cx40 [17], causing slow action potential (AP) propagation, which is a key phenotypic hallmark of SAN. As a consequence, Tbx18 was chosen by Kapoor et al. to induce biopacemaker in neonatal rat VMs [18]. It was the first time applying only one single gene to directly convert cardiomyocytes to pacemaker cells. The converted VMs, called induced SANs, became smaller, thin, and tapered, acquiring the exact morphological characteristics of SAN cells. What is more, the automatic electrical phenotype was also similar to that of SAN pacemaker cells.

In 2014, Hu et al. created a biopacemaker in large animal heart [19], which was effective for up to 14 days. They transduced the gene encoding human Tbx18 into porcine ventricular cardiomyocytes by adenovirus vector. The pacemaker activity, emanating from the injection site, was observed even when the heart was completely blocked, indicating that the induced SAN was successfully working. Furthermore, the increase of arrhythmias was not observed in the biopacemaker heart. The results suggested that somatic reprogramming might be a viable strategy to create biopacemaker, increasing the possibility of clinical translation.

The temporary biopacemaker activity has been observed in bioexperiments, however, which were always expensive, time-consuming, and difficult to make. As a consequence, in this study, we aimed to create a 2D computational model of pacemaker from the human ventricle to simulate the biopacemaker. The reduction of *I*_K1_ could induce automaticity of VMs [20–22]. Therefore, we achieved the single biopacemaker cell by depressing the *I*_K1_ of human ventricular endocardial myocyte. As the automaticity and correlative currents had been analyzed [23] in previous studies, we then investigated the driving force by simulating whether one induced pacemaker cell could drive one VM. Next, the working 2D pacemaker model was developed to investigate the driving force and the number of pacing cells needed for successful pacing. In particular, the importance of the conduction pattern was examined. Finally, the pseudo-ECG was computed to evaluate the function of the induced biopacemaker.

## 2. Methods

### 2.1. Ventricular Sheet Data

**Figure 1:**
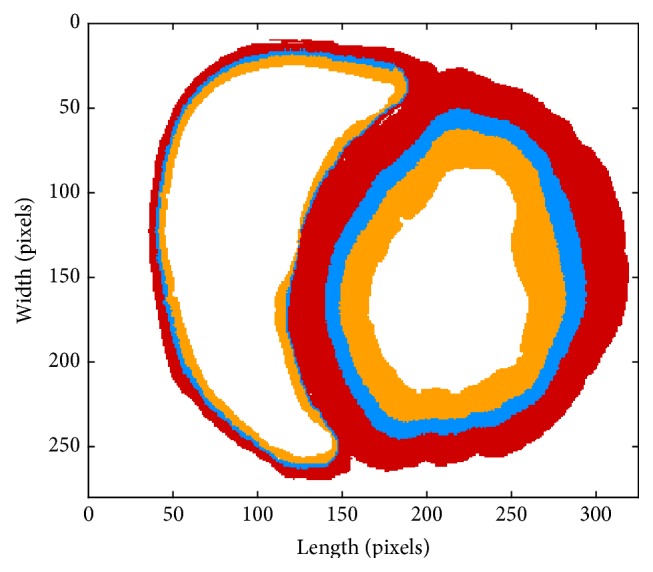
The human ventricular slice. The orange area: the endocardium; the blue region: the midmyocardial layer; and the red space: epicardium.

Our 2D simulation was based on a human ventricular slice, where a pixel, called one computing element, represented an area of size 0.33 mm × 0.33 mm. The slice is shown in Figure 1, where the left part belongs to the right ventricle and the right is the left ventricular tissue. The orange area is the endocardium, the blue region the midmyocardial layer, and the red the epicardium. There are 11343 pixels in endocardium, 6565 in midmyocardial layer, and 16229 in epicardium, respectively, in the slice.

### 2.2. Pacemaker Setting

**Figure 2:**
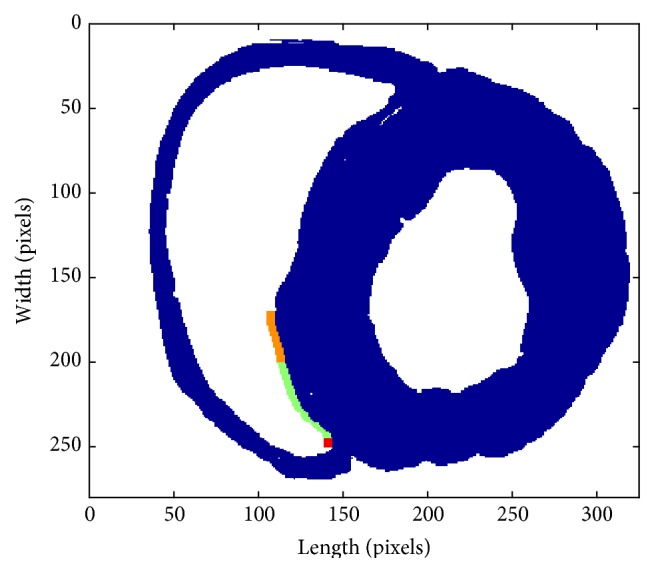
The pacemaker setting. The orange region is the pacemaker, 10 mm long, containing 150 computing elements. The green and red area is the Purkinje fiber, 50 computing elements in width and 5 computing elements in length. The red region consists of 25 computing elements, through which the excitation propagates from the pacemaker to VMs.

According to the experiment in [15], the pacemaker activity conducted to the ventricle through the His-Purkinje system; as a consequence, in the study, the pacemaker was designed as in Figure 2.

The blue area is the original ventricular tissue, and the orange area is the pacemaking region which consists of 5 computing elements in length (from left to right) and 30 computing elements in width (from up to down). In other words, there are only 150 pacemaker computing elements for the whole slice which contains more than 30,000 computing elements.

The automatic rhythm of the pacemaker cells was created from endocardial myocytes induced by depressing *I*_K1_. The green and red area consists of Purkinje fiber cells. Both the orange and the green regions are set electrically insulating from the VMs. The electrical excitation could only propagate through the red region to VMs. The whole Purkinje fiber is 16.5 mm long containing 250 computing elements.

As a comparison, the other pacemaker was designed by simply increasing the number of pacemaking cells, which was discussed in Section 3.2.

### 2.3. Models of VMs and Purkinje Fiber Cells

The well-established TNNP 2006 model of human ventricular cells [24] was used in this study. Briefly, the TNNP single cell model and 2D tissue model for simulating wave propagation are given in (1) and (2), respectively:

(1) dVdt=−Iion+IstimCm,

(2) ∂V∂t=−Iion+IstimCm+DΔV.

In both of the equations,

(3) Iion=INa+IK1+Ito+IKr+IKs+ICaL+INaCa+INaK+IpK+IpCa+IbCa+IbNa,

where *V* is the transmembrane potential; *I*_ion_ is the sum of all the transmembrane ion currents; *I*_stim_ is the external stimulus current and is set to 0 in the paper; *C*_*m*_ is membrane capacitance per unit surface area; *D* is the diffusion tensor describing the conductivity of the tissue, which is 0.00154 cm^2^/ms; Δ is the Laplace operator. *I*_Ks_, *I*_Kr_, and *I*_to_ are the outward slow, rapid, and transient and rectifier potassium currents, respectively. In simulations, except for *G*_K1_, the maximal conductance of *I*_K1_, all other parameters were kept the same as those in the original model. As simulation results suggested that the automaticity of the TNNP cell model disappeared when *G*_K1_ > 2.2 nS/pF, *G*_K1_ was set to 0.05 nS/pF to create biopacemakers in this study.

The Purkinje fiber cell model used in this study was developed by Stewart et al. [25].

### 2.4. Pseudo-ECG Computing

To simulate ECG, one of the virtual electrodes was placed at (*x*_0_, *y*_0_), which was (330, 160) in the study, and the other one was at infinity. The pseudo-ECG was calculated as follows [26]:

(4) $$\begin{array}{c} ECG=\int \frac{\sigma \nabla V_{m}\cdot \overset{⃑}{r}}{r^{3}}dV, \end{array}$$

where *σ* is a constant; ∇ is the gradient operator; *V*_*m*_ is the transmembrane potential; $\overset{⃑}{r}=(x-x_{0},y-y_{0})$ is the vector from the electrode to the point (*x*, *y*) in the tissue; *V* is the area of the virtual tissue; *r* = ((*x* − *x*_0_)^2^ + (*y* − *y*_0_)^2^)^1/2^ is the distance from point (*x*, *y*) to the electrode (*x*_0_, *y*_0_).

Using the Forward Euler method, the pseudo-ECG formulation could be discretized as follows:

(5) ECG=−σ∬1r3x−x0dVmdx+y−y0dVmdydx dy=−σ∑∑1x−x02+y−y023/2·x−x0·Vmx+Δx,y−Vmx−Δx,y2Δx+y−y0·Vmx,y+Δy−Vmx,y−Δy2Δy·ΔxΔy.

In 2D simulation, the time step *dt* was set to 0.02 ms and space step *dh* was 0.33 mm; and all the 2D simulation time was no less than 600,000 ms in order to get a stable state.

## 3. Results

In this section, we first investigated the automaticity of single induced pacemaker cell and then showed the results of one-drives-one model. At last, the 2D pacemaker was designed and the electrical excitation propagation was analyzed.

### 3.1. One-Drives-One Model

**Figure 3:**
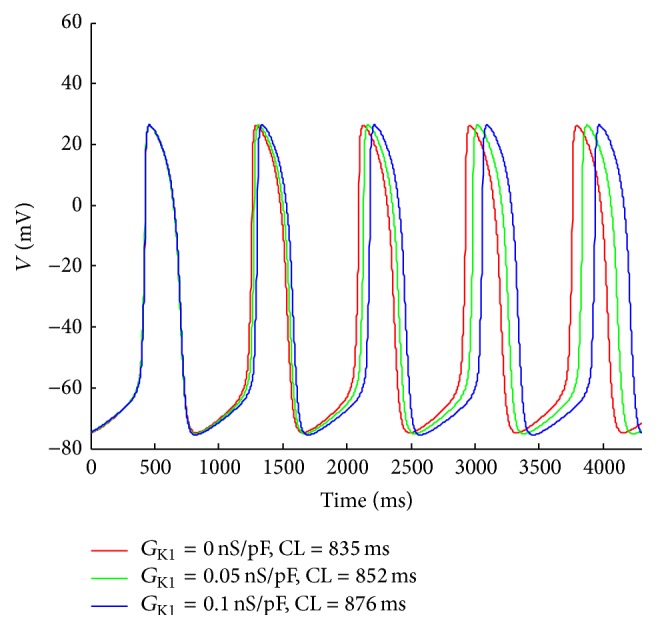
Effects of depressing *I*_K1_ on automaticity of VMs. The red curve describes the APs for *G*_K1_ = 0 nS/pF, which means that *I*_K1_ is completely blocked. The green and blue curves are APs for *G*_K1_ = 0.05 nS/pF and for *G*_K1_ = 0.1 nS/pF, respectively.

Depressing *I*_K1_ by modulating the maximal conductance *G*_K1_ could induce automaticity of VMs. The more the *I*_K1_ was suppressed, the stronger autorhythmicity the myocytes displayed. In Figure 3, we could clearly observe that the cycle length (CL) of automatic AP is obviously decreasing with reducing *G*_K1_, which indicates the enhancement of automaticity with the decline of *I*_K1_.

In bioexperiments, it was usually difficult to block *I*_K1_ completely; therefore, in our study, we chose *G*_K1_ = 0.05 nS/pF for the following simulations. We found that, after 500,000 ms, the single automatic cell (AC), that is, the pacemaker cell used in the present paper, kept in stable state with a cycle length of 852 ms. However, the average cycle length between 70,000 ms and 80,000 ms was about 740 ms. The difference was more than 100 ms. Therefore, the simulation time for 2D slice was set to no less than 600,000 ms to obtain a stable state.

Connecting the two cells with normal diffusion tensor, we examined whether one strong and robust AC could drive only one VM. The result showed that one myocyte could not be driven by one AC. According to Xie et al. [27] and Zhang et al. [28, 29], in 1D strand, the number of contiguous ACs to trigger a propagating action potential was about 70 to 90. In this study, we found that at least 6 ACs were needed to trigger one single VM. However, the process was very slow; it cost more than 800,000 ms to make the first pacing. If so, was a suitable size of ACs able to drive the normal ventricular slice?

### 3.2. 2D Model

**Figure 4:**
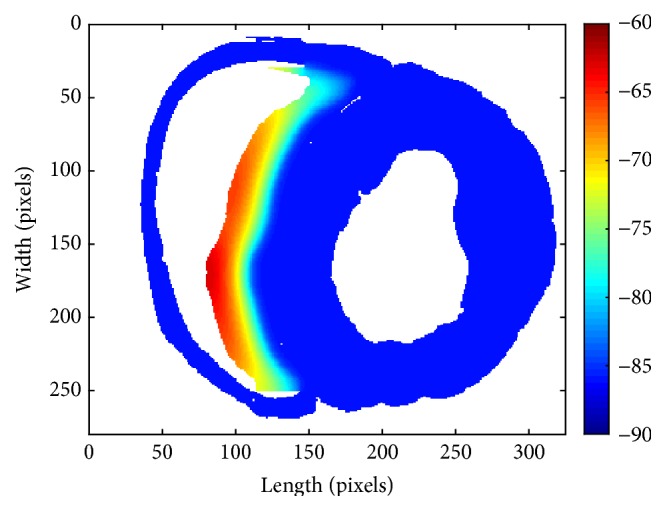
The stalemate for excitation propagation with more than 6600 ACs. The color bar represents the voltage, whose unit is mV. The region in warm color is the ACs whose maximal voltage is less than −60 mV.

In the 2D model, the ACs were firstly directly connected to ventricular slice. We studied the relationship between driving capability and the quantity of ACs, finding that autorhythmic pacemaker activity of the ACs was depressed by the surrounding VMs even under the condition that there were plenty of ACs, which might already lose the biological meaning. As shown in Figure 4, the automatic region and the quiescent myocytes keep in a stalemate in spite of more than 6600 automatic computing elements. Though the surrounding VM cells could not completely depolarize and further fire the excitation propagation, their maximal potential augmented as the quantity of ACs increased. With the continuous increase of ACs, when there were 6820 automatic computing elements, the pacemaker would work; however, it was biologically meaningless.

The effective and reasonable pacemaker might not be created by simply increasing the number of the ACs. The ACs failed to pace because of the suppression from the adjacent VMs. In fact, for native SAN, the salient gap junction proteins made a lower single-channel conductance [30–32]. These shielded the SAN from the hyperpolarization environment of atrial tissue [33]. Accordingly, in the simulation, the electric excitation of ACs was set not directly electric to the ventricular slice, only through the path from Purkinje fiber as shown in Figure 2. In this way, the created pacemaker could be protected from the serious depression of ventricular quiescent cells and obtained a stronger driving capability.

**Figure 5:**
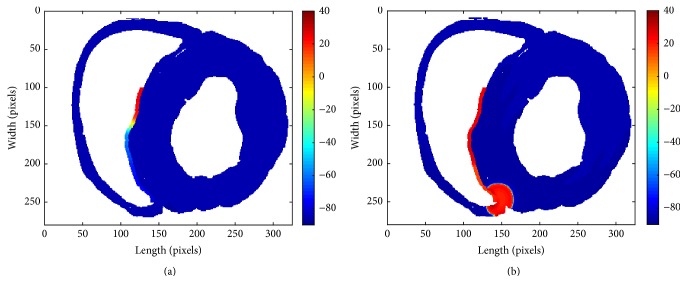
Two snapshots of the successful excitation propagation induced by biopacemaker. (a) The state in the beginning of the pacing. (b) Electrical excitation propagates to the ventricle.

In the beginning, we set up 500 automatic computing elements which were 5 in length and 100 in width along the right edge of interventricular septum, observing that the automatic rhythm was fast and the ventricular tissue could be driven easily.  Figure 5 demonstrates two snapshots of the state of pacemaker firing (a) and excitation propagation (b).

Keeping the length unchanged, the pacemaker was simulated with its width reduced by a step of 5 computing elements. The activities of pacemakers with different width were listed in Table 1, where the unit of the width was computing element. The results showed that the pacemaker worked well with only 125 computing elements. To obtain a small enough and meanwhile strong enough pacemaker, the final size in our study was 5 in length and 30 in width at last, which was shown in Figure 2.

**Figure 6:**
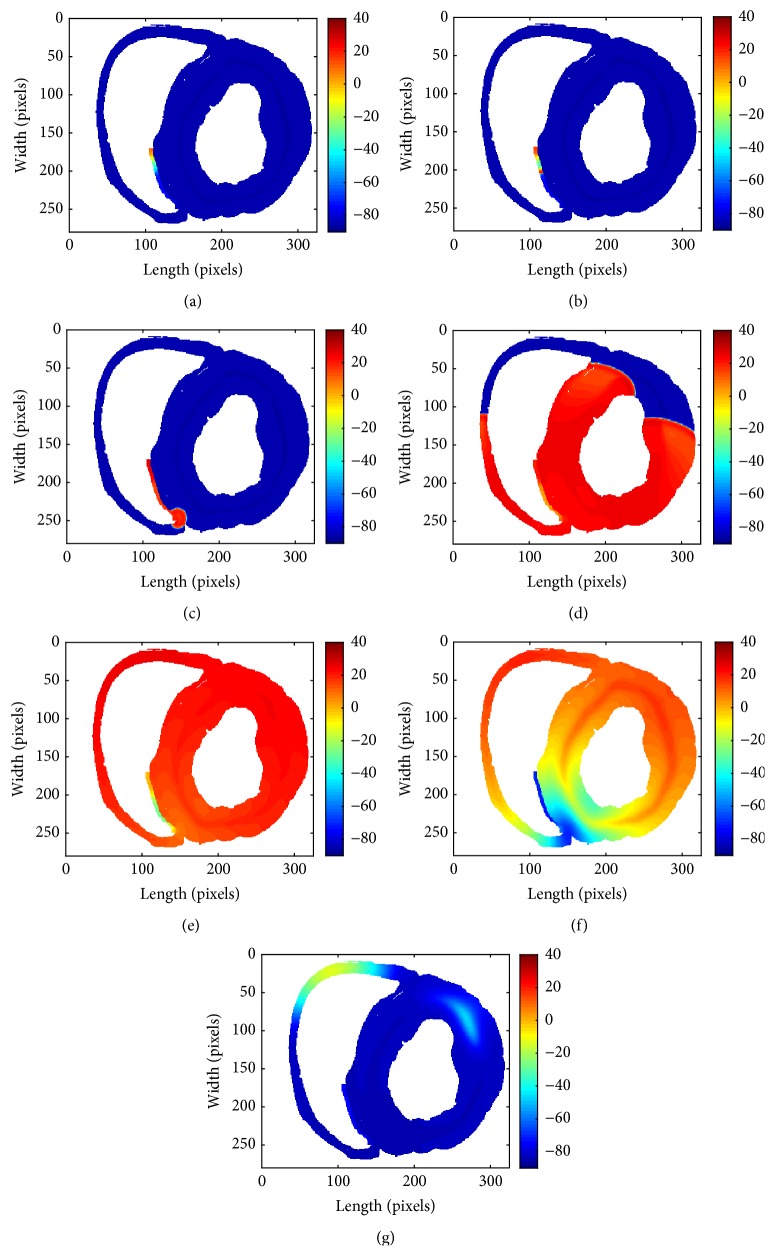
Snapshots of excitation wave emerging and propagating across the ventricular slice. (a) *t* = 890,673 ms; the excitation is emerging. (b) *t* = 890,675 ms; the wave is conducting to Purkinje fiber. (c) *t* = 890,700 ms; the wave is propagating to VMs. (d) *t* = 890,800 ms; the excitation is propagating in the slice. (e) *t* = 890,900 ms; a slow repolarization state of the tissue. (f) *t* = 891,000 ms; a rapid repolarization state of the slice. (g) *t* = 891,120 ms, the end of the repolarization.

Without external stimulation, all the electric excitation was generated by the created pacemaker automatically. The pacemaker started the first pacing in no more than 300 ms and then kept working robustly and periodically.  Figure 6 shows the snapshots of the generation of excitation in the pacemaker and propagation across the ventricular tissue at varying timings. The whole propagation process was shown in Video S1 in the Supplementary Material available online at http://dx.doi.org/10.1155/2016/3830682.

Figure 6 demonstrates that the effective pacemaker was successfully created. In particular, from Figure 6(b), we could observe that the amplitude of APs of the Purkinje fiber cells was larger than those of adjacent ACs. That was in that the velocity of rapid depolarization of the Purkinje fiber cells was faster than that of ACs while the threshold voltage was lower. The fiber cells produced upstrokes once the automatic excitation reached the threshold. As a result, the fiber cells immediately fired AP and spread away.

**Figure 7:**
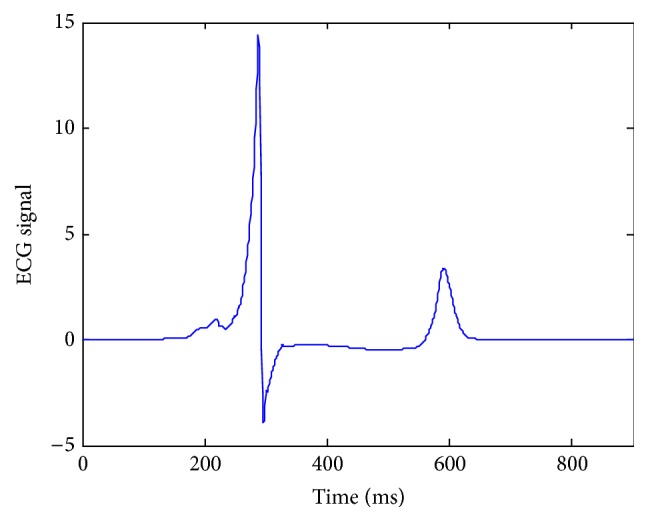
Simulated time course of pseudo-ECG in response to the conduction of excitation wave in the ventricular tissue.

In order to study the global function of the created pacemaker for the ventricular tissue, a pseudo-ECG was calculated. The result was shown in Figure 7. The simulated ECG showed typical features of normal ECG with positive QRS and T waves, which indicated that the created pacemaker played an effective and important role in the ventricular slice.

**Figure 8:**
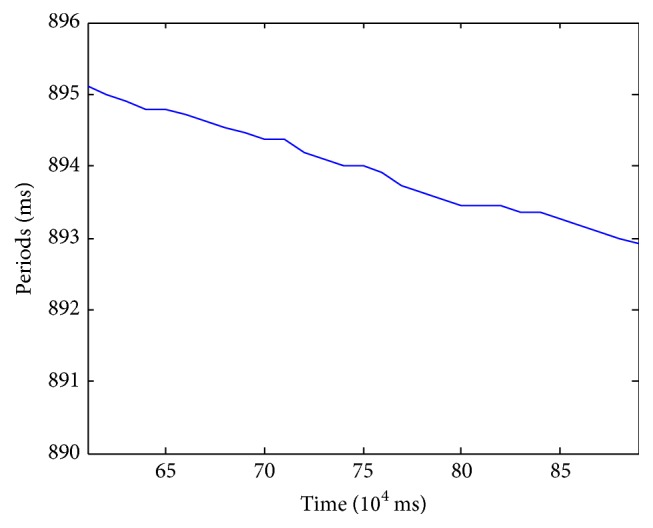
Average periods calculated for the pacemaker per 10,000 ms.

The average cycle lengths of the pacemaker were recorded from 600,000 ms to 900,000 ms. We calculated the average periods every 10,000 ms (Figure 8).

The average periods were around 894 ms in tissue versus 852 ms for single AC, indicating a difference of 42 ms. However, the average periods trended down about 2.2 ms every 5 s. Accordingly, we speculated that the pacemaker might pace with similar period to that of corresponding single pacemaker cell after 96 minutes (5,760,000 ms), which would cost more than a month for simulation.

**Figure 9:**
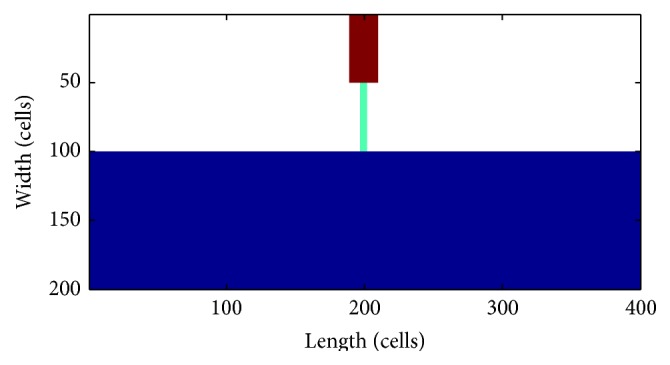
The idealized tissue. The brown region: pacemaker; the green strip: Purkinje fiber; the blue area: VMs. The scales represent cells.

So, we verified the speculation in an idealized tissue which was 400 cells in length and 100 cells in width, shown in Figure 9, where the Purkinje is 7.5 mm long and the pacemaker contains 1000 cells. And we recorded the average periods in every 10,000 ms between 500,000 ms and 600,000 ms. The periods distributed among 851.09 ms and 851.30 ms, which fitted the period (852 ms) of single AC well. The guess could be verified to an extent although it might not be sufficient.

## 4. Discussion

Many experimental studies have been done about biopacemaker. However, as far as we know, there are no corresponding computing models built to simulate the biological characters of the biopacemakers. In the study, utilizing the TNNP06 model of single human ventricle cell [24], we first developed the 2D biopacemaker model and simulated the propagation of the electrical excitation from pacemaker to the whole 2D human ventricular tissue. We examined the stability of the pacemaker and investigated its driving capability, finding the suitable size and reasonable pattern for robust pacing and driving the surrounding quiescent cardiomyocytes.

Qualitatively, the successful pacing of the pacemaker designed in Figure 2 was validated in the previous experimental study [15], where the pacemaker was induced in the atrioventricular junction region and the electrical excitation was conducted through the His-Purkinje system. However, in fact and in some experiments, the biopacemaker could also be induced in other places of the ventricle, where the pacemaker may not be close to the Purkinje system. Thereafter, what will shield the pacemaker from the hyperpolarization environment of ventricular tissue and guarantee the normal pacing? Referencing to the native SAN, the decreased coupling, leading to high intercellular electrical resistance, plays an important part in the pacing. Xie et al. validated that the 6.25-fold decrease in coupling, causing anisotropy and isotropy, reduced the number of ACs required by 2.48- to 2.5-fold [27]. What is more, they got the number of contiguous ACs required to trigger APs for 1D, 2D, and 3D tissue, which gave much inspiration in the development of biopacemaker. As a consequence, the uncoupling between pacing cells, which also has been observed and validated in the biopacemaker [17], will be taken into consideration in our future work, especially in 2D and 3D simulation.

What is more, this study did not include *I*_*f*_, as its role remains a debate [34–37]. However, *I*_*f*_ may be of great significance to the genuine pacemaker [38], which is also a crucial concern in our subsequent research. Nevertheless, there are only one set of experimental data and one model about human *I*_*f*_ [39, 40]. The lack of data of human *I*_*f*_ is one critical reason why the *I*_*f*_ was not introduced. In this study, based on the mathematical analysis [20, 21], we mainly focused on, in particular, the role of *I*_K1_ for the pacemaker activity of human ventricle. In the following study, the role of *I*_*f*_ will be taken into account, according to experimental data recorded in the rodent and other species [41–43].

After all, there is a long way to go to achieve the biopacemaker. However, to create biopacemakers from ventricle, there is a clear direction: depressing *I*_K1_, increasing *I*_*f*_, and reducing cell coupling. In the paper, we focus on the function of depressing *I*_K1_ and the importance of electrical isolation of ACs. In the future work, *I*_*f*_ and cell coupling will mainly be taken into consideration.

In conclusion, a 2D model of biopacemaker was developed in the study. The simulation results suggest that the rhythm of the pacemaker is similar to that of the single cell at final stable state. And the driving force of biopacemaker is closely related to the conduction pattern. This study could provide guidance and advice for future biopacemaker design.

## Supplementary Material

The propagation process of the automatic electrical excitation in about three continuous periods. The time is from 890418ms to 8903100ms.

## Figures and Tables

**Table 1:** Activities of pacemakers with different width.

Width	100	95	90	⋯	40	35	30	25	20	15

Work or not	Yes	Yes	Yes	Yes	Yes	Yes	Yes	Yes	Not	Not

## Abbreviations

*I*_K1_: Inward-rectifier K^+^ current
SAN: Sinoatrial node
VM: Ventricular myocyte
AP: Action potential
AC: Automatic cell
*I*_*f*_: Funny current
CL: Cycle length.
